# Nde1 and Ndel1: Outstanding Mysteries in Dynein-Mediated Transport

**DOI:** 10.3389/fcell.2022.871935

**Published:** 2022-04-12

**Authors:** Sharon R. Garrott, John P. Gillies, Morgan E. DeSantis

**Affiliations:** ^1^ Department of Biological Chemistry, University of Michigan, Ann Arbor, MI, United States; ^2^ Department of Molecular, Cellular, and Developmental Biology, University of Michigan, Ann Arbor, MI, United States

**Keywords:** NDE1, NDEL1, Lis1, cytoplasmic dynein 1, microtubule, motor protein

## Abstract

Cytoplasmic dynein-1 (dynein) is the primary microtubule minus-end directed molecular motor in most eukaryotes. As such, dynein has a broad array of functions that range from driving retrograde-directed cargo trafficking to forming and focusing the mitotic spindle. Dynein does not function in isolation. Instead, a network of regulatory proteins mediate dynein’s interaction with cargo and modulate dynein’s ability to engage with and move on the microtubule track. A flurry of research over the past decade has revealed the function and mechanism of many of dynein’s regulators, including Lis1, dynactin, and a family of proteins called activating adaptors. However, the mechanistic details of two of dynein’s important binding partners, the paralogs Nde1 and Ndel1, have remained elusive. While genetic studies have firmly established Nde1/Ndel1 as players in the dynein transport pathway, the nature of how they regulate dynein activity is unknown. In this review, we will compare Ndel1 and Nde1 with a focus on discerning if the proteins are functionally redundant, outline the data that places Nde1/Ndel1 in the dynein transport pathway, and explore the literature supporting and opposing the predominant hypothesis about Nde1/Ndel1’s molecular effect on dynein activity.

## Introduction

The microtubule-associated motor proteins, cytoplasmic dynein-1 (dynein) and kinesins, promote many types of cellular movements and facilitate intracellular organization in eukaryotes. Dynein and kinesin motors engage with cellular cargos and move along microtubules to power cargo movements that ultimately underlie the spatial and temporal organization of the eukaryotic cytoplasm. Each motor moves in opposite directions on the polar microtubule track: dynein moves in a retrograde fashion toward the microtubule minus-end and nearly all kinesins move in an anterograde manner towards the microtubule plus-end (one exception is the kinesin-14 family, which move towards the minus-end of microtubules (She and Yang, 2017)). Cellular cargos trafficked by dynein and kinesins include, but are not limited to, membrane-bound vesicles, organelles, mRNAs, proteins, and viruses that hijack the motor machinery (Kardon and Vale, 2009; Reck-Peterson et al., 2018). Dynein and kinesins also provide the power to build and separate the mitotic spindle. Dynein localized to the spindle pole body and cortex promotes spindle focusing and alignment (Heald et al., 1996; Gaglio et al., 1997). Dynein at the kinetochore promotes the transition from metaphase to anaphase by trafficking mitotic-spindle checkpoint proteins away from the kinetochore to facilitate progression through metaphase (Vaughan, 2012).

Although both dynein and kinesins use ATP hydrolysis to move along the microtubule track, their evolutionary origins are distinct. Gene duplication and divergence gave rise to a superfamily of kinesins, with each motor specialized for a specific function (Hirokawa and Noda, 2008; Hirokawa et al., 2009). In stark contrast, only a single dynein motor traffics cargo in the cytoplasm and builds the mitotic spindle (Pfister et al., 2006). How dynein can recognize and transport such a diverse array of cargo is only beginning to become clear: a host of dynein regulatory proteins facilitate access to cargo and promote diverse dynein activities.

**FIGURE 1:**
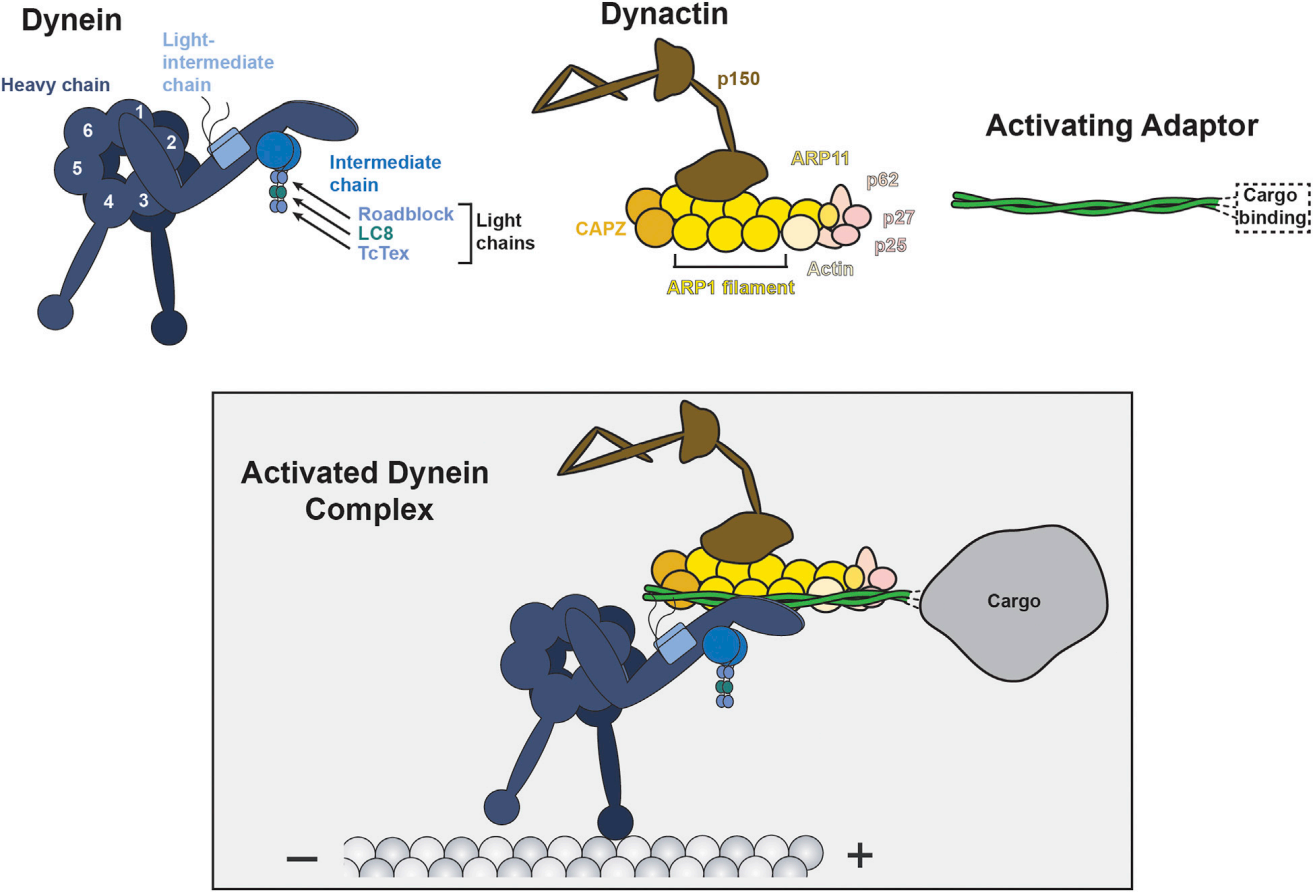
Components of the activated dynein complex. The activated dynein complex (boxed) is the active and processive motile complex driven by dynein motor activity. The activated dynein complex is comprised of dynein (blue), dynactin (yellow/brown/pink), and an activating adaptor (green). Dynein is comprised of six dimeric subunits: heavy chain, intermediate chain, light-intermediate chain, and three distinct light chains. The ATPase motor is contained in the heavy chain and is comprised of six separate ATPase Associated with various cellular Activities (AAA) domains (numbered 1–6), three of which (AAA1, AAA3, and AAA4) are capable of actively hydrolyzing ATP. Dynein’s C-terminal tail extends from AAA6, behind the motor ring, and is not shown in this schematic. Dynactin is a 23-polypeptide complex, with some key subunits indicated. All known activating adaptors are coiled coils, bind dynein and dynactin via domains in the N-terminus, and engage with cargos via domains in the C-terminus (reviewed recently (Reck-Peterson et al., 2018)).

Over the past 20 years, it has become increasingly apparent that regulation of dynein motility is integral to dynein function. Dynein’s affinity for the microtubule track, ability to move processively, or ability to interact with cargoes are regulated by dynein binding proteins (Kardon and Vale, 2009; Cianfrocco et al., 2015). Unlike many kinesins, which are inherently processive, mammalian dynein must bind to at least two additional regulatory partners to achieve processive movement on microtubules. The multi-subunit complex, dynactin, and one of a class of proteins called activating adaptors convert dynein into a processive motor and together form what we will refer to as the *activated dynein complex* (McKenney et al., 2014; Schlager et al., 2014) (Figure 1). In addition to promoting dynein activation, each activating adaptor links dynein to specific subsets of cargo (reviewed recently (Reck-Peterson et al., 2018)). Lis1, a protein best known for association with the developmental disease lissencephaly, is an additional dynein regulator that seems to have two functions: Lis1 modulates dynein’s affinity for microtubules *in vitro* and promotes dynein’s association with dynactin and an activating adaptor (McKenney et al., 2010; Huang et al., 2012; Reiner and Sapir, 2013; Baumbach et al., 2017; DeSantis et al., 2017; Elshenawy et al., 2020; Htet et al., 2020). Together, dynactin, activating adaptors, and Lis1 are three out of four of dynein’s proposed “ubiquitous” regulators, termed such as they are likely involved in all of dynein’s cellular functions (Kardon and Vale, 2009). The fourth ubiquitous regulator designation is shared by the paralogs Nde1 and Ndel1. While there is strong evidence that Nde1 and Ndel1 operate within the dynein transport pathway, the molecular functions of Nde1/Ndel1 remain poorly understood. In this review we will compare Nde1 and Ndel1’s proposed functions, highlight data that places both proteins in the dynein transport pathway, and discuss hypotheses about Nde1/Ndel1’s molecular mechanism.

## Nde1 and Ndel1 are Critical to Development

**FIGURE 2:**
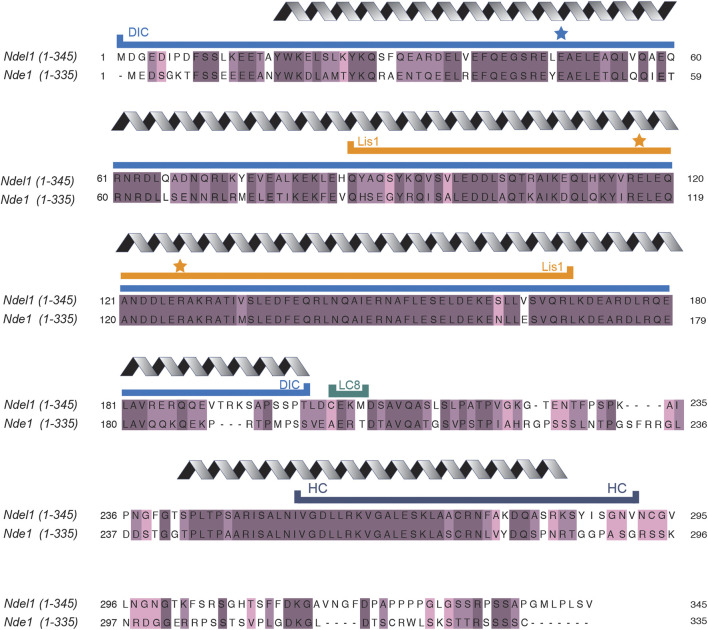
Ndel1 and Nde1 sequence alignments. Sequence alignments of human Ndel1 and Nde1 were performed with Clustal Omega (Sievers et al., 2011). Dark purple indicates residues that are fully conserved, medium purple indicates amino acids that are well-conserved with respect to side chain properties, light purple indicates residues that are weakly conserved. Grey coil above sequence indicates regions that are predicted or observed to be coiled-coils (Derewenda et al., 2007; Ye et al., 2020). All other regions not marked with grey coil are predicted to be random coil or disordered. Minimal regions required to bind dynein intermediate chain (blue line labeled with DIC), Lis1 (orange line labeled with Lis1), dynein light chain 8 (teal line labeled with LC8), and dynein heavy chain (dark blue line labeled with HC) are indicated. Blue star indicates residues that, when mutated, ablate binding to dynein intermediate chain (Derewenda et al., 2007; Wang and Zheng, 2011). Orange stars indicate residues that, when mutated, ablate binding to Lis1 (Wang and Zheng, 2011; Żyłkiewicz et al., 2011; Wang et al., 2013).

Nde1/Ndel1 (previously referred to as NudE/Nudel) are conserved from yeast to human. Budding yeast and filamentous fungi contain just one *Nde1/Ndel1*-type gene, while higher eukaryotes, including zebrafish and *Xenopus,* have two distinct genes (Drerup et al., 2007; Ma et al., 2009). Although very similar in sequence (human Nde1 and Ndel1 are 56% identical and 69% similar) (Figure 2) (Altschul et al., 2005; Sievers et al., 2011), Nde1 and Ndel1 are expressed differently. While both human proteins are expressed in all tissues, Nde1 expression is highest for a few months immediately after fertilization and then tapers down to a relatively low level in most tissues (Cardoso-Moreira et al., 2019). Ndel1 expression is high and relatively constant throughout life (Bradshaw et al., 2013; Cardoso-Moreira et al., 2019). Consistent with an important role throughout the entire developmental timeline, *Ndel1* knockout mice are not viable (Sasaki et al., 2005), while *Nde1* knockout mice survive (Feng and Walsh, 2004). Despite differences in viability, both proteins appear to regulate the same process in neurodevelopment: *Ndel1* conditional knockout mice and *Nde1* knockout mice both display cortical thinning resulting from neuron progenitor migration defects (Feng and Walsh, 2004; Youn et al., 2009; Soto-Perez et al., 2020).

Nde1 and, to a lesser extent, Ndel1 are associated with neurodevelopmental diseases. Biallelic *Nde1* mutation or deletion is associated with severe brain malformations, including microcephaly, microlissencephaly, and microhydrancephaly (Alkuraya et al., 2011; Bakircioglu et al., 2011; Guven et al., 2012; Lipka et al., 2013; Paciorkowski et al., 2013; Abdel‐Hamid et al., 2019). Copy number variation of the locus containing *Nde1* is associated with epilepsy, autism, and intellectual disability (Ullmann et al., 2007; Hannes et al., 2009; Heinzen et al., 2010; Mefford et al., 2010; Tropeano et al., 2013). Schizophrenia is associated with duplication or deletion of the gene locus containing *Nde1* and *Ndel1* (Kirov et al., 2009; Ingason et al., 2011; Malhotra and Sebat, 2012; Rees et al., 2014; Johnstone et al., 2015). It is unclear if *Nde1*’s stronger link to disease phenotypes is due to differences in the two proteins’ functions or simply due to differences in expression during development. Given that *Ndel1* knockout is embryonic lethal, substantial copy number variation or mutation of *Ndel1* may not be permissive to development (Sasaki et al., 2005).

## The Molecular Function of Nde1/Ndel1 Requires Further Study

The molecular mechanism of Nde1/Ndel1 remains ill-defined. There are two primary reasons for the uncertainty surrounding Nde1/Ndel1’s molecular function. First, experiments probing Nde1/Ndel1 function have been conducted in many different model organisms, cell culture systems, and *in vitro,* and have yielded contradictory findings. For example, mammalian Nde1 appears to negatively regulate dynein’s microtubule binding affinity, while the yeast Nde1/Ndel1 homolog does not (Yamada et al., 2008; McKenney et al., 2010; McKenney et al., 2011; Torisawa et al., 2011; Huang et al., 2012). It is not clear if contradictory results arise from bona fide functional differences across species, differences in experimental systems, or reflect functional plasticity inherent in Nde1/Ndel1. Secondly, Nde1/Ndel1, in conjunction with dynein, regulate multiple and diverse cellular processes ranging from promoting spindle assembly during division to driving nuclear oscillations in dividing neural progenitor cells. It is not clear if Nde1/Ndel1 fulfill the same role during each process they support.

Despite relatively high homology and a shared importance in neurodevelopment, in some contexts, Nde1 and Ndel1 seem to regulate different cellular events (Vergnolle and Taylor, 2007; Doobin et al., 2016). The extent to which Nde1 and Ndel1 have divergent or redundant functionality has not been firmly established. Both proteins are subject to extensive and often unique post-translational modifications (reviewed in (Bradshaw et al., 2013)). In cases where the proteins receive the same post-translational modification on the same amino acid, the modification can have different effects on the association of Nde1 and Ndel1 with dynein. For example, while both Nde1 and Ndel1 can be palmitoylated, this modification negatively regulates Ndel1 binding to dynein, but does not affect Nde1’s interaction with dynein (Shmueli et al., 2010). Finally, while both proteins bind directly to dynein and some of its regulatory proteins (discussed in detail below), Nde1 and Ndel1 each interact with a unique subset of other proteins (reviewed in (Bradshaw et al., 2013)), supporting the idea that Nde1 and Ndel1 may enable dynein to access different cellular pathways. Below we will discuss Nde1 and Ndel1’s cellular function, with specific emphasis on work that probes Nde1/Ndel1’s function with respect to dynein. We will also highlight, whenever possible, evidence that Nde1 and Ndel1 serve similar or disparate cellular roles.

## Cellular Evidence for Nde1/Ndel1’s Role as a Dynein Regulator

Dynein is a remarkably multifunctional motor protein. As the primary retrograde microtubule motor in most eukaryotes, dynein must engage with hundreds of different types of cargo and provide the power to reshape and focus the mitotic spindle. There is evidence that Nde1 and Ndel1 are involved in most dynein-dependent processes (Simões et al., 2018).

### Nuclear Positioning

**FIGURE 3:**
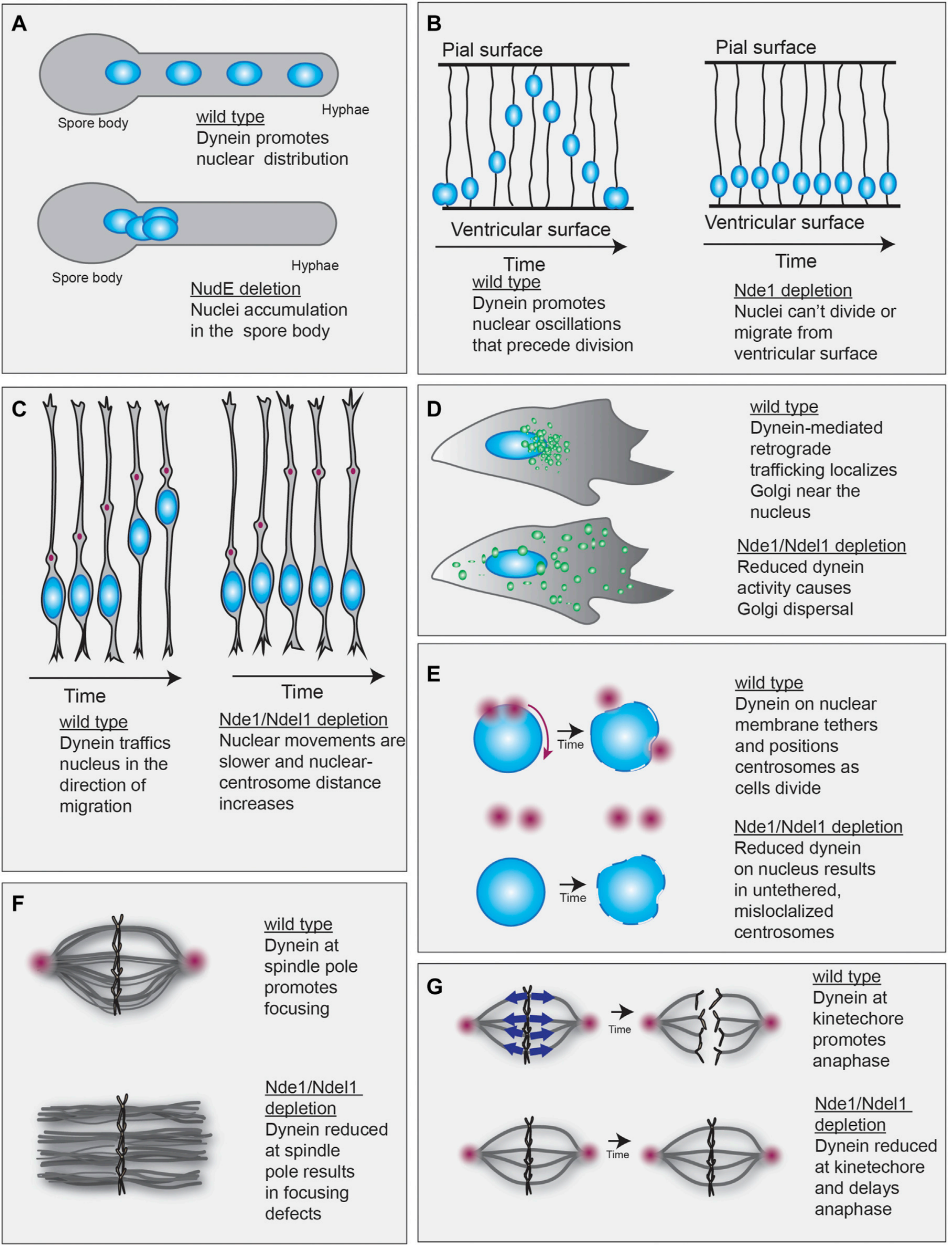
Evidence of a role for Nde1/Ndel1 in dynein-mediated transport. **(A)** Dynein supports localization of nucleus (blue spheres) along the hyphae in filamentous fungi (top). NudE or ro-11 depletion causes nuclei to accumulate in the spore body (bottom) (Bruno et al., 1996; Minke et al., 1999; Efimov and Morris, 2000). **(B)** Dynein drives the ventricular-directed movement of nuclei in neural progenitors. These oscillations are required for cell division (left). Nde1 depletion prevents nuclear oscillatory motions, likely by inhibiting mitosis (right) (Doobin et al., 2016). **(C)** After division, neural progenitor cells migrate away from the ventricular zone. During this type of migration, the centrosome (red) is oriented in the direction of migration and its forward movement precedes nucleokinesis (left). Nde1/Ndel1 depletion prevents dynein-driven nucleokinesis, resulting in slow migration velocities and increased distance between the centrosome and nucleus (right) (Shu et al., 2004; Youn et al., 2009; Doobin et al., 2016). **(D)** Retrograde trafficking mediated by dynein promotes the juxta-nuclear localization of the Golgi (green) (top). Nde1/Ndel1 depletion results in Golgi dispersal, suggesting dynein transport defects (bottom) (Liang et al., 2004; Lam et al., 2010). **(E)** Dynein anchored at the nuclear envelope helps position and anchor centrosomes during cell division (top). Nde1/Ndel1 depletion reduces dynein at the nuclear envelope, which leads to impaired centrosome localization (bottom). **(F)** Dynein promotes spindle focusing (top). Depletion of Nde1 and Ndel1 in *xenopus* egg extracts generates spindle focusing defects (bottom) (Wang and Zheng, 2011; Wang et al., 2013). **(G)** Dynein localized to the kinetochore primarily functions to inactivate the mitotic spindle checkpoint to promote transition to anaphase. Dynein (blue arrows) accomplishes the checkpoint inactivation by trafficking check-point proteins away from the kinetochore and towards the spindle pole, where microtubule minus-ends are clustered (red sphere) (left). Nde1 depletion reduces dynein localization at the kinetochore resulting in prolonged mitosis (right) (Vergnolle and Taylor, 2007).

One of the first observable functions of dynein was to promote proper nuclear positioning in fungi (Xiang et al., 1994; Plamann et al., 1994; Eshel et al., 1993; Li et al., 1993). A Nde1/Ndel1-type protein was first identified in mutagenesis screens seeking to identify proteins that, like dynein, promote nuclear positioning in the filamentous fungi *N. crassa* and *A. nidulans* (Minke et al., 1999; Efimov and Morris, 2000; Bruno et al., 1996). Mutants of the Nde1/Ndel1-type proteins identified (called ro-11 in *N. crassa* and NudE in *A. nidulans*) display defective hyphal growth and nuclear distribution defects, with nuclei failing to migrate into the hyphae and accumulating closer to the spore body (Figure 3A). This phenotype placed ro-11 and NudE in the dynein regulatory pathway and was the first evidence that a Nde1/Ndel1-type protein may work in conjunction with dynein to promote nuclear distribution (Li et al., 1993; Plamann et al., 1994; Xiang et al., 1994; Xiang et al., 1995).

Nde1 and Ndel1 are implicated in nuclear positioning in other organisms, as well. Nde1 promotes the nuclear oscillations that precede radial glial progenitor cell division. This process, called interkinetic nuclear migration (INM), occurs in the ventricular zone of the developing cortex (reviewed in (Bertipaglia et al., 2018)). Although the developmental function of INM is unknown, it is a conserved process (Kishimoto et al., 2013; Leung et al., 2011; Meyer et al., 2011; Tsai et al., 2010; Azizi et al., 2020). Dynein, dynactin, and Lis1 promote the apical-directed nuclear movements during INM (Tsai et al., 2010; Tsai et al., 2005; Tsai et al., 2007; Del Bene et al., 2008). Nde1 depletion also impairs apical-directed nuclear migration, suggesting a shared function with dynein transport machinery (Doobin et al., 2016) (Figure 3B). Interestingly, Ndel1 depletion does not effect INM, supporting distinct functionality of these proteins during this process (Doobin et al., 2016).

After INM, neural progenitor cells migrate out of the ventricular zone to the outer strata of the brain. During this process, dynein-dependent nucleokinesis occurs, where dynein powers the transport of the nucleus in the direction of migration. Lis1 depletion impairs nucleokinesis during neuron migration, supporting its role in dynein activation (Youn et al., 2009; Tsai et al., 2007). Consistent with this, Ndel1 depletion increases the centrosome to nucleus distance in migrating mouse neurons, which is a hallmark of impaired nucleokinesis (Youn et al., 2009; Shu et al., 2004). Similarly, depletion of Nde1 impairs migration velocity, which is consistent with what is seen upon Ndel1 depletion (Doobin et al., 2016; Shu et al., 2004) (Figure 3C). Furthermore, exogenous Nde1 expression can rescue Ndel1 depletion defects during migration, supporting an overlapping role during migratory nucleokinesis (Doobin et al., 2016).

### Cargo Trafficking

One of dynein’s primary functions in somatic cells is cargo trafficking. In rat dorsal root ganglia, Ndel1 depletion has little effect on retrograde cargo trafficking flux, but when combined with Lis1 depletion, both retrograde and anterograde trafficking is severely impaired (Pandey and Smith, 2011). This result is similar to when the dynein heavy chain is depleted (Pandey and Smith, 2011). A common hallmark of dynein malfunction is Golgi dispersal (Burkhardt et al., 1997; Corthésy-Theulaz et al., 1992). In HeLa or HEK293T cells, Nde1 or Ndel1 depletion results in mild Golgi dispersal (Liang et al., 2004; Lam et al., 2010). Significant dispersal occurs when both Nde1 and Ndel1 are depleted simultaneously (Lam et al., 2010) (Figure 3D). Exogenous expression of either Nde1 or Ndel1 can rescue the dispersal phenotype, suggesting an overlapping function of the paralogs (Lam et al., 2010). Similarly, microinjection of a function-blocking anti-Nde1/Ndel1 antibody causes rapid dispersal of acidic organelles, suggesting acute inhibition of retrograde, dynein-mediated trafficking (Yi et al., 2011). In COS1 cells, Nde1 and Ndel1 depletion reduced microtubule-motor-dependent movement of lipid droplets. Specifically, overall movement and run lengths of lipid droplets were reduced upon simultaneous Nde1 and Ndel1 depletion (Reddy et al., 2016). Together, this body of evidence supports a role for both Nde1 and Ndel1 in cargo trafficking.

### Mitotic Functions

Some of the most substantial evidence that Nde1/Ndel1 promote dynein activity comes from studies probing dynein activity during cell division. Dynein promotes multiple steps of cell division (Dwivedi and Sharma, 2018). First, dynein activity at the nuclear pore complex promotes nuclear envelope breakdown that precedes division (Salina et al., 2002; Li et al., 2010). Second, dynein facilitates duplicated centrosome positioning and helps assemble and focus the mitotic spindle (Robinson et al., 1999; Sitaram et al., 2012; Titus and Wadsworth, 2012). Finally, dynein promotes the transition from metaphase to anaphase by transporting checkpoint proteins away from kinetochores (Gassmann et al., 2010; Howell et al., 2001). There is evidence that Nde1/Ndel1 support nearly all of dynein’s mitotic dynein functions. Both Nde1 and Ndel1 drive dynein localization to the nuclear envelope to promote nuclear envelope breakdown and positioning of centrosomes, (Figure 3E), promote spindle formation and focusing (Figure 3F), and facilitate dynein localization at the kinetochore in prometaphase (Figure 3G) (Liang et al., 2007; Mori et al., 2007; Stehman et al., 2007; Vergnolle and Taylor, 2007; Hebbar et al., 2008; Wainman et al., 2009; Bolhy et al., 2011; Wang and Zheng, 2011; Żyłkiewicz et al., 2011; Monda and Cheeseman, 2018; Wynne and Vallee, 2018). Although Nde1 and Ndel1 seem capable of fulfilling similar roles during cell division, Nde1 drives dynein localization at kinetochores to a greater extent than Ndel1 (Vergnolle and Taylor, 2007). Additionally, Nde1 depletion impairs cell division more than Ndel1 depletion in neural progenitors (Doobin et al., 2016). Together, these results suggest that Nde1 and Ndel1 may have subtly distinct roles during cell division.

### Additional Functions of Nde1/Ndel1

Though this review is focused on Nde1/Ndel1’s role in dynein activity, it is important to emphasize that Nde1 and Ndel1 have additional cellular functions, some of which may be dynein-independent. One of the best characterized interaction partners of Nde1 and Ndel1 is the protein Disrupted-In-Schizophrenia-1 (DISC1) (Porteous et al., 2006). DISC1 is involved in the pathology of psychiatric disorders including schizophrenia, bipolar disorder, and depression (Korth, 2009; Soares et al., 2011). DISC1 is part of a large interaction network with proteins that are involved in several key signaling pathways (Millar et al., 2005; Soares et al., 2011; Bradshaw and Porteous, 2012). The protein binding interaction between Nde1, Ndel1, and DISC1 is well-established and the genetic interaction between *Nde1/Ndel1* and *DISC1* is associated with an increased risk for schizophrenia (Morris, 2003; Ozeki et al., 2003; Brandon et al., 2004; Burdick et al., 2008; Ye et al., 2020). However, the molecular outcome of Nde1/Ndel1 binding to DISC1 is not yet fully elucidated. There is evidence that DISC1 may promote Nde1’s localization to kinetochores, which may in turn promote dynein localization (Ye et al., 2020). However, more work is required to determine if Nde1 or Ndel1 function to modulate DISC1 activity, to promote DISC1 access to the dynein transport pathway, or if the role that Nde1 and Ndel1 play in DISC1 biology represent a largely dynein-independent process.

Nde1 plays a role in safeguarding the genome of neural progenitor cells against DNA damage (Houlihan and Feng, 2014). During DNA replication in S-phase, *Nde1* homozygous deletion knockout mice (Nde1^_^/^_^) accrue double stranded DNA breaks that result in elevated levels of apoptosis (Houlihan and Feng, 2014). It is hypothesized that nuclear-localized Nde1 interacts with the chromatin associated proteins, like cohesin and the remodeler SNF2h, to aid in DNA remodeling during replication (Houlihan and Feng, 2014). Given the absence of dynein from the nucleus during S-phase, it is unlikely that dynein is involved in this Nde1 functionality.

Nde1 is negative regulator of primary cilia length, with excess expression of Nde1 promoting short cilia (Kim et al., 2011; Maskey et al., 2015). To modulate ciliary length, Nde1 protein levels are tightly controlled during G1/G0 and CDK5 phosphorylation of Nde1 ultimately promotes its proteasomal degradation (Maskey et al., 2015). The role of dynein in this Nde1 activity is unknown.

Ndel1 is a serine oligopeptidase and can cleave unstructured peptides (Hayashi et al., 2000; Hayashi et al., 2005). The peptidase activity is inhibited by DISC1 binding, suggesting that Ndel1 peptidase activity is under tight regulation (Hayashi et al., 2005). How or if Ndel1’s oligopeptidase activity effects Ndel1-mediated dynein regulation remains to be determined.

## The Molecular Determinants of Nde1/Ndel1’s Regulation of Dynein

Despite evidence that Nde1 and Ndel1 function in the dynein transport pathway, their molecular function with respect to dynein activity remains elusive. Several *in vitro* studies have used purified proteins to investigate the functional outcome of Ndel1 and Nde1’s interaction with dynein or its regulatory partners. However, it is still not clear how or if Nde1/Ndel1 directly affect dynein motility. It is worth noting that the majority of *in vitro* work designed to understand how Nde1/Ndel1 influence dynein motility was conducted before the discovery that dynein requires both dynactin and an activating adaptor to achieve true processive motility (McKenney et al., 2014; Schlager et al., 2014). Below, we will outline the data that describe how Nde1 or Ndel1 interact with dynein and its regulatory proteins, then discuss the evidence for and against the current model for how Nde1/Ndel1 modulate dynein activity.

### Nde1/Ndel1 Structure

Nde1 and Ndel1 have similar secondary structures, with both proteins containing long stretches of coiled-coil and random coil (Figure 2). Both proteins are dimers and may assemble into tetramers under some conditions (Derewenda et al., 2007; Soares et al., 2012). There is evidence that Nde1 and Ndel1 can form heterodimers with each other, as well, although it is not clear what proportion of the Nde1 and Ndel1 population are homodimers vs heterodimers *in vivo* (Bradshaw et al., 2009).

### Nde1/Ndel1 Bind to Lis1

Nde1 and Ndel1 and Lis1 binding is well-described and well-characterized (McKenney et al., 2010; Efimov and Morris, 2000; Wang and Zheng, 2011; Derewenda et al., 2007; Tarricone et al., 2004; Feng et al., 2000). The binding interface for Lis1 is contained within amino acids 85–169 of Ndel1 (a region that is highly conserved with Nde1) (Derewenda et al., 2007). The interaction of Ndel1 with Lis1 is relatively strong, with a high nanomolar dissociation constant (Tarricone et al., 2004). On Ndel1, the interaction has been mapped with high precision: both Ndel1^E116A^ and Ndel1^R127A^ completely abolish binding to Lis1, as demonstrated with human and *Xenopus* proteins (indicated by orange stars in Figure 2) (Derewenda et al., 2007; Wang and Zheng, 2011). The interaction with Nde1/Ndel1 has been mapped to one side of the Lis1 beta-propeller, and yeast two-hybrid experiments have implicated the Lis1 residues Lis1^S169^ and Lis1^H149^ as key mediators of the interaction with Nde1 and Ndel1 (Feng et al., 2000; Tarricone et al., 2004).

### Nde1/Ndel1 Bind to Dynein

**FIGURE 4:**
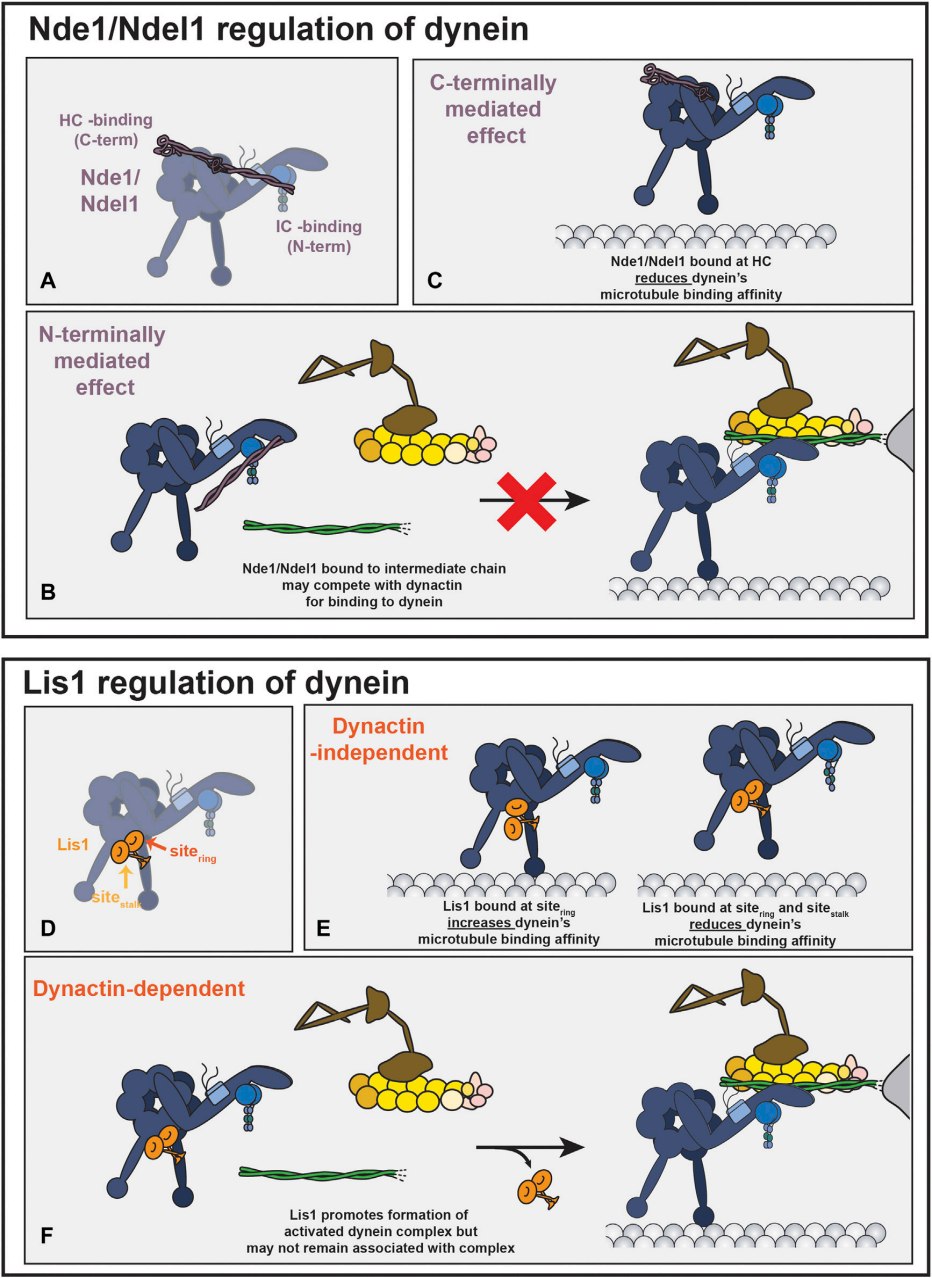
Nde1/Ndel1 and Lis1 regulation of dynein. **(A)** Nde1/Ndel1, shown in purple, binds to up to three places on the dynein motor. The binding with the intermediate chain (IC) is mediated by Nde1/Ndel1’s N-terminus and binding with the heavy chain (HC) is mediated by Ndel1’s C-terminus (Liang et al., 2004; Sasaki et al., 2005; McKenney et al., 2011; Wang and Zheng, 2011; Żyłkiewicz et al., 2011; Nyarko et al., 2012; Wang et al., 2013). Binding between Nde1/Ndel1 and dynein’s LC8 chain is not shown (Stehman et al., 2007; McKenney et al., 2011). Although this schematic shows Nde1/Ndel1 binding to both the intermediate chain and the heavy chain simultaneously, this has not been observed directly. **(B)** By binding competitively with dynactin for dynein’s intermediate chain, Nde1/Ndel1’s N-terminus could potentially inhibit activated dynein complex formation (McKenney et al., 2011; Nyarko et al., 2012; Jie et al., 2017). **(C)** The interaction between Nde1/Ndel1’s C-terminal fragment and dynein reduces dynein’s interaction with the microtubule track (Torisawa et al., 2011). **(D)** Lis1 binds to two places on the dynein motor domain. Site_ring_ (indicated with dark orange arrow) is located on the motor domain between AAA3 and AAA4. Site_stalk_, indicated with a light orange arrow, is located on the stalk insertion in AAA4. Two binding sites have been observed with yeast and human proteins (DeSantis et al., 2017; Htet et al., 2020). **(E)** Lis1 binding at just site_ring_ increases dynein’s affinity for microtubules. Lis1 binding at both sites reduces dynein’s microtubule binding affinity. The low-affinity regulator state, however, has only been observed with yeast proteins. **(F)** Lis1, likely by binding to both sites, promotes dynein’s association with dynactin and an activating adaptor (Gutierrez et al., 2017; Elshenawy et al., 2020; Htet et al., 2020). Lis1 likely promotes association with dynactin and activating adaptor by disfavoring a conformation of dynein that has a reduced affinity for dynactin (not shown) (Zhang et al., 2017; Qiu et al., 2019; Elshenawy et al., 2020; Htet et al., 2020; Marzo et al., 2020). Once the activated dynein complex is formed, it is not clear if Lis1 remains bound to the activated dynein complex, as some studies have observed that Lis1 dissociates after complex formation and others have observed that Lis1 remains bound (Baumbach et al., 2017; Gutierrez et al., 2017; Jha et al., 2017; Elshenawy et al., 2020; Htet et al., 2020). Lis1 also promotes formation of activated dynein complexes with two dynein dimers (not shown) (Elshenawy et al., 2020; Htet et al., 2020). While not illustrated in this figure, the tether model suggests that Nde1/Ndel1 associated with dynein IC can promote Lis1 binding on the dynein motor domain.

There is evidence that Nde1 and Ndel1 bind directly to multiple sites on the dynein motor (Figure 4A). One of the binding sites for Nde1/Ndel1 is on the dynein intermediate chain (McKenney et al., 2011; Wang and Zheng, 2011; Żyłkiewicz et al., 2011; Wang et al., 2013; Nyarko et al., 2012). Nde1 and Ndel1 constructs containing the first ∼200 amino acids are sufficient to bind dynein intermediate chain robustly, and the point mutant, Ndel1^E48A^, inhibits binding (marked by a blue star in Figure 2) (Wang and Zheng, 2011; Żyłkiewicz et al., 2011; Wang et al., 2013). The binding site for Nde1/Ndel1 is within the first 40 amino acids of dynein intermediate chain (McKenney et al., 2011; Nyarko et al., 2012). This interaction is well-supported as much of the work validating it was performed with pure proteins (McKenney et al., 2011; Wang and Zheng, 2011; Nyarko et al., 2012; Wang et al., 2013), allowing for detailed affinity measurement showing a dissociation constant in the low micromolar range (Nyarko et al., 2012; Wang et al., 2013).

In addition to dynein intermediate chain, Ndel1 is also reported to bind to dynein heavy chain. Yeast two-hybrid assays and immunoprecipitations from HEK293 cell lysate support a binding interface between Ndel1 amino acids 256–291 and dynein’s heavy chain, potentially near one of dynein’s primary ATPase domains (AAA1) or the C-terminus (Liang et al., 2004; Sasaki et al., 2000). Despite relatively high conservation with Ndel1 in the proposed binding site, purified Nde1 and dynein motor domain do not bind *in vitro* (Figure 2) (McKenney et al., 2010).

Finally, Nde1 (amino acids 200–203) binds directly to the dynein light chain, LC8 (Stehman et al., 2007; McKenney et al., 2011). Intriguingly, these residues are not completely conserved between Nde1 and Ndel1, highlighting another potential difference in dynein association between the two paralogs.

### Nde1 and Ndel1 May Bind Directly to Some Dynein Cargo

Nde1 and Ndel1 bind directly to GTP-Rab9A with low micromolar affinity (Zhang et al., 2022). The interaction between Rab9a and Nde1/Ndel1 likely promotes dynein’s association to Rab9-positive late endosomes (Zhang et al., 2022). This finding suggest that Nde1/Ndel1 may function as cargo adaptors in some contexts and directly link the dynein transport machinery to certain cargos.

### Nde1/Ndel1 Compete with Dynactin for Binding to Dynein Intermediate Chain

In addition to binding to Nde1 and Ndel1, dynein intermediate chain interacts with the p150 (also called p150^Glued^) subunit in dynactin (Nyarko et al., 2012; Jie et al., 2017; Karki and Holzbaur, 1995; Siglin et al., 2013; Vaughan and Vallee, 1995; Paschal et al., 1993). p150 and Nde1/Ndel1 associate with dynein intermediate chain via overlapping, yet not identical binding sites and Nde1 appears to bind competitively with p150 (McKenney et al., 2011; Nyarko et al., 2012; Jie et al., 2017). The competitive nature of these interactions suggest that Nde1/Ndel1 may affect or regulate the formation of the activated dynein complex (Figure 4B). However, the regions of p150 and dynein intermediate chain that interact are not resolved in the structures of the activated dynein complex, making it difficult to conclude how Nde1/Ndel1 and p150 competition for dynein intermediate chain would affect dynein activation (Urnavicius et al., 2015; Grotjahn et al., 2018; Urnavicius et al., 2018).

### Nde1/Ndel1 May Modulate Dynein’s Microtubule-Binding Affinity

Several studies show that mammalian Nde1 and Ndel1 negatively regulate dynein’s microtubule-binding affinity (McKenney et al., 2010; McKenney et al., 2011; Yamada et al., 2008; Torisawa et al., 2011). C-terminal truncations of Ndel1 are sufficient to recapitulate the reduction in dynein’s microtubule binding affinity (Torisawa et al., 2011) (Figure 4C). The mechanistic basis or physiological relevance for the change in microtubule-binding affinity is unknown. Nde1/Ndel1 from budding yeast (called Ndl1) lacks the C-terminal domain and does not modulate yeast dynein’s microtubule-binding affinity (Huang et al., 2012), suggesting that the ability for Nde1 and Ndel1 to regulate dynein’s microtubule-binding affinity is not shared by all organisms.

### Support For and Against the Model That Nde1/Ndel1 Tethers Lis1 to Dynein

Lis1 is a catalytically inactive subunit of the platelet-activating factor acetylhydrolase (PAF-AH) and a well-studied dynein regulatory protein (reviewed recently (Markus et al., 2020)). Several studies over the last decade have revealed how Lis1 regulates dynein (Huang et al., 2012; DeSantis et al., 2017; Htet et al., 2020; Elshenawy et al., 2020; Baumbach et al., 2017; Splinter et al., 2012; Marzo et al., 2020; Qiu et al., 2019; Gutierrez et al., 2017; Jha et al., 2017) (Figures 4D–F). Lis1 interaction with dynein has two general outcomes, which are not necessarily mutually exclusive: first, in the absence of dynactin and an activating adaptor, Lis1 modulates dynein’s apparent affinity for microtubules (Huang et al., 2012; DeSantis et al., 2017; Htet et al., 2020; McKenney et al., 2010; Toropova et al., 2014) (Figure 4E). Secondly, in the presence of dynactin and an activating adaptor, Lis1 increases the formation of the activated dynein complex by disfavoring dynein’s autoinhibited conformation (Htet et al., 2020; Elshenawy et al., 2020; Marzo et al., 2020; Qiu et al., 2019; Zhang et al., 2017) (Figure 4F). Lis1 also promotes the formation of activated dynein complexes that contain two dynein dimers, which results in an activated dynein complex that moves with an elevated velocity (Htet et al., 2020; Elshenawy et al., 2020). Lis1 binds to two different sites on the dynein motor, although it is currently unclear what contribution each binding site has to each Lis1 function (Figure 4D) (DeSantis et al., 2017; Htet et al., 2020; Gillies et al., 2022). One of the cellular outcomes of Lis1’s interaction with dynein is to promote dynein’s association with dynactin and cargo-bound activating adaptors (Coquelle et al., 2002; Lee et al., 2003; Sheeman et al., 2003; Markus et al., 2009; Splinter et al., 2012). It is currently unclear how Nde1/Ndel1 fit into this schema.

The prevailing model for Nde1/Ndel1’s function in the dynein transport pathway is to increase Lis1’s association with dynein. Nde1/Ndel1 are hypothesized to tether dynein and Lis1 together (Tarricone et al., 2004; McKenney et al., 2010; Wang and Zheng, 2011; Żyłkiewicz et al., 2011). In this model, Nde1/Ndel1 act as an amplifier of the Lis1 effect by increasing the effective affinity between Lis1 and dynein. There is significant evidence that supports the tether model. In budding yeast, filamentous fungi, *Xenopus* egg extract, several cell culture lines, and in migrating neurons, the deleterious effects of Nde1/Ndel1 depletion can be rescued by Lis1 overexpression, which is consistent with a model where Nde1/Ndel1’s purpose is to tether Lis1 to dynein (Efimov, 2003; Shu et al., 2004; Li et al., 2005; Lam et al., 2010; Wang and Zheng, 2011; Moon et al., 2014). Overexpression of the PAF-AH catalytic subunits, α1 and α2, inactivates dynein, likely by competing for Lis1 binding (Ding et al., 2009). Ndel1 overexpression rescues the α1 and α2-induced dynein inactivation, suggesting that Ndel1 can promote Lis1-dynein association (Ding et al., 2009). In *A. nidulans*, mutations in dynein that promote its ability to form the activated dynein complex partially rescue the effect of NudE depletion, bolstering the idea that NudE works in conjunction with Lis1 to activate dynein (Qiu et al., 2019). In further support of a tethering function, experiments conducted with purified mammalian proteins have shown that Nde1 increases the association of Lis1 and dynein (McKenney et al., 2011). Consistent with a tethering role, with purified *S. cerevisiae* proteins, Ndl1 decreases the amount of Lis1 (called Pac1 in yeast) required to slow the velocity with which dynein moves on microtubules, suggesting that Ndl1 acts to increase the effective concentration of Pac1 (Huang et al., 2012). (It is important to note that yeast dynein is processive in the absence of dynactin and an activating adaptor and that Pac1 slows the velocity of walking dynein (Reck-Peterson et al., 2006; Huang et al., 2012).) Altogether, there is compelling data that support a role for Nde1/Ndel1 in stabilizing or promoting the interaction with dynein and Lis1 in a way that amplifies Lis1’s effect on dynein.

However, not all studies support a simple tether model of Nde1/Ndel1 function. Lis1 overexpression does not rescue the axon swelling caused by Ndel1 depletion in neurons (Kuijpers et al., 2016). Furthermore, the centrosome localization defect caused by Ndel1 depletion can be rescued by exogenous expression of a Ndel1 mutant that cannot bind Lis1 (Monda and Cheeseman, 2018). Similarly, anaphase onset delays induced by Nde1 depletion are largely rescued by expression of a Nde1 mutant that doesn’t bind Lis1 (Wynne and Vallee, 2018). Importantly, a dynein binding-deficient Nde1 construct does not rescue the anaphase onset delays, supporting a dynein-dependent role for Nde1 during cell cycle progression (Wynne and Vallee, 2018). Additionally, Lis1 mutation and Nde1 mutation are associated with distinct neurodevelopmental diseases. Nde1 mutation is largely associated with microcephaly, while Lis1 mutation is associated with lissencephaly (Lipka et al., 2013; Reiner and Sapir, 2013), suggesting that Lis1 and Nde1/Ndel1 have unique functions during development.

The strongest evidence that opposes the tether model comes from *in vitro* studies with mammalian proteins. The Lis1-mediated increase in dynein’s microtubule-binding affinity is not amplified by the presence of Nde1 or Ndel1 as the tether model predicts. Instead, the decrease in microtubule-binding affinity caused by Nde1 and Ndel1 and the increase caused by Lis1 is additive (Yamada et al., 2008; McKenney et al., 2010; Torisawa et al., 2011). Additionally, Lis1 promotes the association of dynein with dynactin, while Nde1/Ndel1 may compete with dynactin for dynein binding (McKenney et al., 2011; Nyarko et al., 2012; Splinter et al., 2012; Elshenawy et al., 2020; Htet et al., 2020).

What could be the function of Nde1/Ndel1 if they promote Lis1-dynein binding while simultaneously inhibiting Lis1’s ability to increase dynein’s microtubule-binding affinity or recruit dynactin? If Nde1/Ndel1 are not functioning as a simple tether, what is their function? One possibility is that Nde1/Ndel1 promote Lis1 association with dynein in a step that is temporally separated from Lis1-mediated dynein activation. Indeed, during INM, two separate pools of dynein are recruited to the nuclear envelope. The activating adaptor BicD2 recruits one pool of dynein to the nuclear envelope (Hu et al., 2013). Dynein recruited in this stage is likely bound to both BicD2 and dynactin in the activated dynein complex (Splinter et al., 2010). A second pool of dynein is sequentially recruited to the nuclear envelope in a Nde1/Ndel1 dependent manner (Hu et al., 2013). Given that Lis1 depletion effects INM at multiple stages, it is likely that Lis1 is involved in recruiting both pools of dynein (Tsai et al., 2005). This may suggest that Nde1/Ndel1’s function in the dynein transport pathway isn’t to promote binding of dynein to dynactin and an activating adaptor, as Lis1 does, but to fulfill some other, unknown role that is temporally separated from Lis1-mediated activation. Since dynein and Lis1 localization are often dependent on Nde1/Ndel1’s proper localization, we hypothesize that Nde1/Ndel1 may function as scaffolds to colocalize dynein and Lis1 while preventing Lis1 from acting on dynein. Because Nde1/Ndel1 may bind competitively with dynactin and reduce dynein’s microtubule binding affinity, we postulate that Nde1/Ndel1 may promote dynein and Lis1 association yet keep dynein and Lis1 in an inactive conformation until some unknown event triggers Lis1-induced dynein activation, dynactin and activating adaptor association, and Nde1/Ndel1 release.

Finally, both Nde1 and Ndel1 are highly phosphorylated by numerous kinases (reviewed in (Bradshaw et al., 2013)). Most exploration of the effect of phosphorylation on Nde1/Ndel1 activity comes from cell-based studies. These studies have shown that phosphorylation of Nde1 and Ndel1 is critically important to their localization, association with dynein and Lis1, and affects the ability of Nde1 and Ndel1 to promote the dynein-Lis1 interaction (Yan et al., 2003; Hebbar et al., 2008; Bradshaw et al., 2011; Bradshaw et al., 2013; Klinman and Holzbaur, 2015; Klinman et al., 2017; Wynne and Vallee, 2018). For example, phosphorylation by Cdk1 promotes Nde1 localization to the nuclear envelope and kinetochores, while Aurora-A kinase phosphorylation increases Ndel1 localization to centrosomes (Mori et al., 2007; Wynne and Vallee, 2018). Cdk5 phosphorylation of Ndel1 regulates Ndel1’s ability to bind dynein and promotes dynein’s association with microtubules (Hebbar et al., 2008; Klinman and Holzbaur, 2015; Klinman et al., 2017). Cdc2, Erk1, and Erk2 activity increase association between Ndel1 and Lis1 (Yan et al., 2003). PKA phosphorylation of Nde1 increases binding to Lis1 and promotes heterodimerization or tetramerization between Nde1 and Ndel1 (Bradshaw et al., 2011). Altogether, there is significant evidence that, in cells, post-translational modification of Nde1 and Ndel1 alter the mechanistic outcome of Nde1 and Ndel1 binding to dynein and its regulatory partners. However, most *in vitro* studies to date have ignored Nde1/Ndel1 phosphorylation as a variable in determining their molecular activity. This may account for some of the discrepancies observed between Nde1 and Ndel1’s activities in cells and *in vitro*.

There are many outstanding questions about Nde1 and Ndel1 function and interaction with the dynein transport machinery. Some of these questions include: 1) How do Nde1/Ndel1 affect dynein motility in the presence of dynactin, an activating adaptor, and Lis1? 2) How do post-translational modifications alter Nde1 and Ndel1’s ability to interact with dynein and its regulators? 3) Do Nde1 and Ndel1 modulate dynein the same way or do the differences that underlie the proteins’ cellular functions reveal differences in their regulation of dynein? 4) Do Lis1 and Nde1/Ndel1 always function in tandem or are there Lis1-independent functions of Nde1/Ndel1? Recent advances in our understanding of dynein regulation by dynactin and Lis1 required the integration of biochemistry, structural biology, and cell biology studies. A similar approach will be required to develop a unified theory of Nde1 and Ndel1 regulation and to fully unpack Nde1 and Ndel1’s molecular mechanism.
